# Onset of Work-Life Conflict Increases Risk of Subsequent Psychological Distress in the Norwegian Working Population

**DOI:** 10.3390/ijerph192013292

**Published:** 2022-10-14

**Authors:** Andrea Rørvik Marti, Eirik Degerud, Tom Sterud

**Affiliations:** Department of Occupational Health Surveillance, National Institute of Occupational Health, 0363 Oslo, Norway

**Keywords:** work-family conflict, work-life balance, anxiety, depression, mental health, occupational health

## Abstract

We aimed to assess whether the onset of work-life conflict is associated with a risk of subsequent onset of psychological distress. Respondents from a randomly drawn cohort of the general Norwegian working population were interviewed in 2009 (T1), 2013 (T2), and 2016 (T3) (gross sample *n* = 13,803). Participants reporting frequent work-life conflict at T1 and/or psychological distress (five-item Hopkins Symptom Checklist mean score ≥ 2) at T2 were excluded to establish a design that allowed us to study the effect of the onset of work-life conflict at T2 on psychological distress at T3. Logistic regression analysis showed that the onset of frequent work-life conflict more than doubled the risk of the onset of psychological distress at T3 (OR = 2.55; 95% CI 1.44–4.51). The analysis of the association between occasional work-life conflict and psychological distress was not conclusive (OR = 1.21; 95% CI 0.77–1.90). No differential effects of sex were observed (log likelihood ratio = 483.7, *p* = 0.92). The calculated population attributable risk (PAR) suggests that 12.3% (95% CI 2.84–22.9%) of psychological distress onset could be attributed to frequent work-life conflict. In conclusion, our results suggest that the onset of frequent work-life conflict has a direct effect on the future risk of developing symptoms of psychological distress in both male and female workers.

## 1. Introduction

The balance between work and family life, or more typically the lack thereof, is referred to as work-family or work-life conflict or interference in the literature [1,2]. Work-life conflict arises because of requirements to meet expectations or demands in both the work and home domains. The concept of work-life conflict is not new and has been discussed in the literature since at least the 1980s. However, as aspects of work and family life change over time, work-life conflict-related issues continue to emerge as important in the world of work. A recent example is the COVID-19 pandemic, which caused sudden and dramatic changes, with millions of people moving from work at their regular employer premises to their own homes [3]. On one hand, telework may positively impact worker well-being, perceived autonomy, and work-life conflict [4,5]. On the other hand, there is concern that the blurring of lines between the work and family domains may negatively impact work-life balance [3]. There is also concern regarding the possible negative impact of work-life conflict on workers’ mental health, which is a significant and increasing cause of both sick leave and long-term labour market exclusion [6,7].

Several prospectively designed studies have examined work-life conflict as a potential cause of poor mental health, but the overall evidence appears to be inconclusive. In a large Japanese cohort, work-life conflict predicted psychological distress in both men and women a year later, even after applying stringent criteria to control for fixed effects [8]. Similarly, a study of Swedish workers showed that work-life conflict was associated with an increased incidence of emotional exhaustion in both genders at a two-year follow-up [9]. A study of Australian parents reported that the onset of work-life conflict was associated with the subsequent onset of mental health problems, while a reduction in work-life conflict over time was associated with a lower incidence of mental health problems [10]. Furthermore, a representative sample of Australian fathers showed that work-life conflict predicted later psychological distress [11]. A study using a random sample of Finnish workers showed that work-life conflict predicted job dissatisfaction and mental health symptoms a year later, with the most pronounced effect in women [12]. There are also prospective studies that fail to find an association between work-life conflict and mental health. In a longitudinal study of two representative samples of the Finnish working population, no associations were found between work-life conflict and psychological well-being at either one- or six-year follow-up [13]. Furthermore, in a study of Norwegian mothers, work-life conflict did not predict burnout and exhaustion after five nor eighteen years of follow-up [14].

In summary, although a predominance of studies reports an association between work-life conflict and measures of psychological distress, there are some unanswered questions. First, the potential bidirectionality, whereby mental health problems could potentially also lead to or intensify work-life conflict [15], has only been specifically addressed in one previous study [10]. Second, some studies [9,12,16] suggest that men and women have different experiences and health consequences as a result of work-life conflict, but the results are not conclusive.

The aim of the present study was to examine whether the onset of work-life conflict predicts subsequent onset of psychological distress in a sample of Norwegian workers. We make use of a prospective longitudinal design to take the temporal aspects of both work-life conflict and mental health problems into account, something that few studies have done. We further tested whether the association differed by sex. Lastly, to indicate the contribution of work-life conflict as a risk factor for psychological distress in the population, we calculated the population attributable risk (PAR).

## 2. Materials and Methods

### 2.1. Study Population and Design

The Survey of Level of Living—Working Conditions is an ongoing nationwide survey of Norwegian residents aged 16–66 years, where data is collected by Statistics Norway every three years, via phone interviews [17,18,19]. In the present study, data from three consecutive surveys were included. The first survey (T1; data collection: June–January 2009/10) resulted in 12,255 interviews (60.9%) out of a gross sample of 20,136 randomly drawn from the population. The second survey (T2; April–January 2013/14) invited the same gross sample to participate and 10,875 responded (53.1%). In the third survey (T3; September–April 2016/17) about two-thirds of the original gross sample were re-invited (*n* = 12,928) due to a planned rotation of the panel selection, and among these 7327 were interviewed (56.7% response rate). In the present study, the 12,928 individuals that were initially selected to participate in all three survey waves made up the source sample. From this sample, we excluded people who did not respond to the questionnaire at either T1, T2, or T3, who were unemployed at T1 or T2, and people with missing values on work-family conflict at T1 or T2 or psychological distress at T2 or T3.

To generate a design that allows us to study the prospective association of the onset of work-life conflict with the development of psychological distress, we used T1 to wash-out individuals with frequent work-life conflict and T2 to measure exposure to new onset work-life conflict and wash-out individuals with baseline psychological distress. Onset of psychological distress was measured at T3, that is, three years after the onset of work-life conflict. This approach was chosen to address two important limitations in the literature. First, by constructing a cohort of workers where no one was exposed to frequent work-family conflict at baseline, we reduce the risk of selection bias that can be introduced if some individuals are more prone to reporting work-family conflict and psychological distress over time, which could contribute to overestimating the association. Second, by studying the onset of psychological distress at follow-up, we reduce the risk of bi-directionality whereby mental health problems could potentially also lead to or intensify work-life conflict.

### 2.2. Measurements

#### 2.2.1. Work-Life Conflict

Work-life conflict was measured with the question: “How often do the demands at your work interfere with your home and family life?” Answer categories were “very seldom or never”, “rather seldom”, “sometimes”, “rather often” or “very often or always”. By combining “very seldom or never” with "rather seldom” and by combining “rather often” with “very often or always”, we created a three-level discrete variable with the categories “no”, “occasional” or “frequent” work-life conflict.

#### 2.2.2. Psychological Distress

Symptoms of depression and anxiety were measured using a 5-item version of the Hopkins Symptoms Checklist (HSCL-5). The 25-item HSCL-25 is a validated, widely used measure of depression and anxiety [20], and the shorter 5-item version was recently validated and found to have good correlation with the original version, as well as good convergence across sex and age groups [21]. Participants were asked to rate the following symptoms of depression and anxiety in the past 14 days on a 4-point scale: (i) feeling fearful; (ii) nervousness or shakiness inside; (iii) feeling hopeless about the future; (iv) feeling blue; and (v) worrying too much about things. An index was computed as the mean of the item scores. A cut-off point for HSCL-5 of ≥ 2.0, which is the cut-off suggested by a previous study that compared the performance of the 5-item version to the 25-item version [22], was used to define cases of mental distress.

#### 2.2.3. Demographic Variables

Information about education level, number of children living at home, and marital status were derived from administrative registry data. Sex, age, and occupation were self-reported during the telephone interview. The occupation was coded by a trained interviewer into a professional title in accordance with the International Standard Classification of Occupations (ISCO-08). Professional titles were categorised into 17 occupational groups using the first and second digits of the ISCO-08 code.

#### 2.2.4. Statistical Analysis

The distribution of work-family conflict and psychological distress at T2 was described according to covariates, and the chi-square test was used to test for differences. The association between the onset of work-life conflict at T2 and the risk of psychological distress at T3 was assessed using logistic regression analysis. Three logistic regression models were run; model 1 was adjusted for age and sex; model 2 was further adjusted for occupation and education level; and model 3 was further adjusted for children under the age of 18 living at home and marital status. Prospective associations between work-life conflict (occasional and frequent) and psychological distress were reported as odds ratios (OR) with 95% confidence intervals (CI). To assess whether the association of work-life conflict and subsequent psychological distress is different for women and men, a version of model 3 run with sex as an interaction term was compared with the original model 3 using the log-likelihood ratio test. All analyses were carried out using the statistical software R v.3.6.1. Statistical significance was accepted at *p* < 0.05.

We calculated the population attributable risk (PAR%) of psychological distress caused by work-life conflict using the formula Pd*((OR-1)/OR), where Pd is the proportion of cases exposed to the risk factor in question. The lower and upper limits of the 95% CI for PAR% were calculated from the general PAR% formula using the lower and upper limits of the 97.5% CI for Pd and OR [23]. The interpretations of PAR estimates are based on the theoretical assumption that the exposure-response relationship is causal.

## 3. Results

### 3.1. Participants and Selection

**Figure 1:**
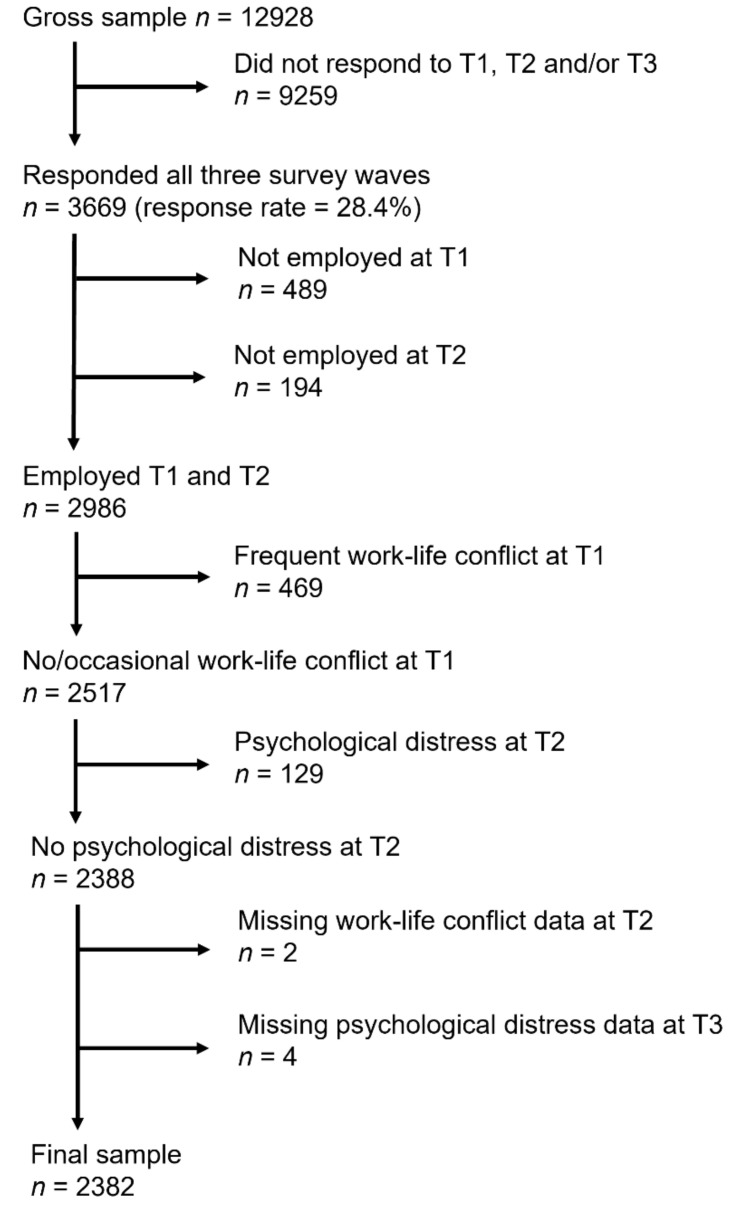
Sample.

The flow of participants from the source population to the final sample is described in Figure 1. In total, 12,928 individuals were invited to participate in all three survey waves. Of these, 9259 did not respond to one or more of the surveys. A total of 683 were excluded due to a lack of employment at T1 or T2. Further, 469 reporting frequent work-life conflict at T1 and 129 reporting psychological distress at T2 were excluded (wash-out criteria). Finally, six participants were excluded due to missing data on relevant variables.

The final sample included in statistical analyses comprised 2382 individuals. To evaluate the degree to which the selection criteria may bias the demographic makeup of the final sample, we present some key numbers for the baseline gross sample and the final sample at T2. At T2, the baseline gross sample comprised 51% men while the final sample comprised 52% men. As for age, the final sample comprised more individuals in the age groups 35–44 and 45–55 as compared to the gross sample (26% vs. 20% and 34% vs. 22%). In contrast, the gross sample comprised more individuals in both the youngest (17–24; 11% vs. 3% and 25–34; 18 vs. 14%) and oldest (55–66; 29% vs. 24%) age groups as compared to the final sample. The final sample had a higher proportion of individuals with university education (52% vs. 36%) and a lower proportion of those with elementary education only (8% vs. 21%). 

### 3.2. Descriptive Statistics

Table 1 shows the number of observations and the prevalence of different levels of work-life conflict according to study variables. Of the 2382 participants included in the final sample, 906, 1219, and 257 reported no, occasional, and frequent work-life conflict at T2, respectively. The prevalence of work-life conflict was similar between the sexes. Work-life conflict was reported more frequently in the age groups 25–34 and 35–44 and among those with university education. Work-life conflict was also more prevalent among married and single participants, as well as among those with children living at home.

### 3.3. Associations between the Onset of Work-Life Conflict and Subsequent Psychological Distress

Table 2 shows that the onset of frequent work-life conflict at T2 was strongly associated with the onset of psychological distress at T3, as compared with no work-life conflict at T2. The association was strengthened after adjustment for occupation and education level, marital status, and children living at home. As for occasional work-life conflict, results were inconclusive, with OR indicative of a small positive association but confidence intervals ranging from a reduction to a near-doubling of risk of psychological distress.

We performed another logistic regression analysis based on model three that included an interaction (sex*work-life conflict) to test whether the association of work-family conflict and psychological distress differed by sex. The model did not differ statistically from the original model three, suggesting that sex did not moderate the association between work-life conflict and psychological distress (log likelihood ratio = −483.7, χ^2^_(2)_ = 0.16, and *p* = 0.92).

### 3.4. Population Attributable Risk

To quantify the potential contribution of work-life conflict to the risk of subsequent psychological distress in the population, we estimated the population attributable risk (PAR). Results of the PAR analysis estimate that 12.3% (95% CI 2.84–22.9%) of psychological distress cases could be attributed to the onset of frequent work-life conflict.

## 4. Discussion

In this three-wave follow-up study derived from a representative sample of Norwegian workers, we observed that the onset of frequent but not occasional work-life conflict was associated with the onset of psychological distress three years later in comparison with no work-life conflict. The results were similar for both men and women. Estimates of population attributable risk suggest that more than one in ten cases of psychological distress could be attributed to the onset of frequent work-life conflict at the population level. Initiatives aimed at achieving a stronger balance between work and family life at the workplace may be utilised to alleviate some of these negative effects.

Several prospective studies have found that work-life conflict is associated with subsequent psychological distress (adjusted for baseline symptoms of distress) [8,9,11,12,24]. Our study adds to this literature by showing that the onset of frequent work-life conflict is associated with the incidence of new cases of psychological distress. With a similar design, one previous study [10] showed that the onset of work-life conflict increased the risk of poor mental health, with the poorest mental health found among those with sustained high levels of work-life conflict, while mental health improved with a reduction of work-life conflict. Taken together, the literature and the present results indicate that there is a directional and possibly causal link from work-life conflict to subsequent psychological distress and poor mental health.

The fact that we did not observe that the association between work-life conflict and psychological distress differed according to sex is partly in conflict with some of the literature. The issue of gender has long been of interest to scholars studying work-life conflict, as it has been suggested that women, to a larger extent than men, are burdened by expectations to meet demands in both work and the home arena (the double burden hypothesis), resulting in health problems and subsequent elevated rates of sickness absence [25]. Some studies also suggest that women may be more vulnerable to the negative effects of work-life conflict than men [16]. However, the literature is not fully consistent. In line with our result, a systematic review was unable to find evidence of the double-burden hypothesis when examining associations between work-life conflict and subsequent sickness absence [25]. Additionally, in one study, work-life conflict was similarly associated with emotional exhaustion in both men and women, while a stronger association was observed with self-rated poor health in women and problem drinking in men [9]. Thus, while work-life conflict may be associated with psychological distress in both sexes, the behavioral manifestations of this may differ between men and women. 

Our study indicated that a substantial proportion of cases of psychological distress could potentially be attributed to work-life conflict. This finding suggests there is considerable potential for the prevention of psychological distress cases by addressing the issue of work-life conflict in the working population. Work-life initiatives typically aim to improve workers’ work-life balance or support their non-work lives through initiatives such as reduced working hours, schedule control, flexible working hours, or telework [26]. The evidence for the efficiency of such initiatives to improve worker mental health has been somewhat limited [27,28], but with the COVID-19 pandemic there has been a surge of interest in the use of telework as a flexible tool to support workers’ work-life balance. Recently, a longitudinal study of a representative sample of British workers showed that the perceived availability of work-life initiatives improved subsequent mental health among both women and men, although the use of such initiatives improved mental well-being among women only [29]. Of the three initiatives examined in the study, telework was found to be the most effective work-life initiative to improve mental health among women [29]. Telework may improve the opportunities to spend time doing non-work-related activities by reducing commute hours and increasing flexibility [30]. However, such initiatives must be implemented with caution as many scholars worry that it can also negatively impact work-life balance by blurring the lines between the work and family spheres, preventing employees from ever fully ‘logging off’, both physically and mentally [3,31]. More research is needed to examine the extent to which telework impacts work-life conflict and determine the appropriate framework within which such initiatives should be implemented.

The present study has some important strengths and weaknesses. Strengths include the prospective design and the randomly drawn national sample. Although the samples of each survey have been shown to be relatively representative, individuals with only elementary education are somewhat underrepresented in the surveys [17,18,19]. Furthermore, the inclusion of only those who participated three times in a row introduced a bias toward an older, more highly educated sample. Thus, the final sample was most representative of individuals who report the highest degree of work-life conflict, and the interpretation of ORs and generalisability of PAR estimates should be limited to this group. We used a validated five-item version of the Hopkins Symptom Checklist (HSCL-5), which is considered an adequate screening tool for depression and anxiety [20,21], but the use of a single-item measure to identify work-life conflict represents a potential limitation. Work-life conflict is a multifaceted concept, and using a single item measure precludes any deeper analyses into the different aspects of this concept, including directionality (work-to-family/life and family/life-to-work), or type of conflict (time-based, strain-based, behaviour-based, energy-based) [2,32]. Furthermore, some literature suggests that psychological distress can induce work-life conflict [15], although other studies find no evidence for this [13,14]. However, in the present study we accounted for psychological distress by washing out individuals with psychological distress at baseline, and this should perhaps minimize the risk of reverse causality. Finally, the validity of the PAR estimate relies on the assumption that the association between the exposure variable and the outcome variable is causal. While our study design and results support the inference of potential causality, we cannot rule out residual confounding related to unmeasured variables. 

## 5. Conclusions

The present study supports the inference that the onset of work-life conflict can influence the risk of developing symptoms of psychological distress in the general working population. However, our analyses did not support the inference that the strength of this association differed according to sex.

## Figures and Tables

**Table 1:** Number of observations and prevalence of work-life conflict according to sex, age, education, occupation, marital status, and children living at home.

	Observations (N)	Work-Life Conflict		
		No	Occasional	Frequent
**Sex**				
Men	1242	38%	51%	11%
Women	1140	38%	52%	10%
Chi squared		Χ^2^_(2)_ = 0.76 *p* = 0.68		
**Age**				
17–24	62	61%	35%	3%
25–34	328	35%	48%	17%
35–44	623	32%	56%	13%
45–54	800	37%	53%	10%
55–66	569	46%	47%	7%
Chi squared		Χ^2^_(8)_ = 58.0, *p* < 0.001		
**Education**				
Elementary	202	51%	40%	10%
Secondary	928	46%	45%	9%
University	1244	30%	58%	12%
Chi squared		Χ^2^_(4)_ = 75.2, *p* < 0.001		
**Occupations (ISCO)**				
Managers (11–13)	228	19%	71%	10%
Hospitality, retail, other service managers (14)	35	29%	54%	17%
Professionals (21, 24–26)	474	30%	54%	16%
Health professionals (22)	191	31%	56%	14%
Teaching professionals (23)	253	27%	61%	12%
Technicians, associate professionals (31, 33–35)	338	38%	55%	7%
Health associate professionals (32)	54	54%	41%	6%
Clerks (41)	48	50%	40%	10%
Customer services clerks (42–44)	87	51%	44%	6%
Personal service workers (51)	71	54%	30%	17%
Sales workers (52)	66	59%	38%	3%
Personal care workers (53)	150	58%	35%	7%
Protective services workers (54)	19	42%	58%	0%
Agricultural/forestry/fishery workers (61–64)	42	36%	48%	17%
Craft and related trades workers (71–75)	178	49%	42%	9%
Plant-/machine operators, assemblers (81–83)	102	57%	34%	9%
Elementary occupations (91–96)	23	61%	39%	0%
Unspecified	23	48%	35%	17%
Chi squared		Χ^2^_(34)_ = 190.8, *p* < 0.001		
**Marital status**				
Married/cohabiting partner	1416	36%	53%	11%
Single	819	41%	48%	11%
Divorced/widowed	147	44%	50%	7%
Chi squared		Χ^2^_(4)_ = 10.8, *p* =0.03		
**Children under 18 living at home**				
No	1268	43%	48%	9%
Yes	1114	32%	55%	13%
Chi squared		Χ^2^_(2)_ = 29.6, *p* < 0.001		

**Table 2:** Logistic regression: Psychological distress at T3 regressed on work-life conflict at T2.

			Model 1		Model 2		Model 3	
**Work-Life Conflict**	***N***	*N* cases (%)	OR§	95%CI	OR§	95%CI	OR§	95%CI
No	906	35 (3.9%)	1.00		1.00		1.00	
Infrequent	1219	52 (4.3%)	1.10	0.71–1.71	1.15	0.74–1.80	1.21	0.77–1.90
Frequent	257	22 (8.6%)	2.24	1.28–3.91	2.32	1.32–4.09	2.55	1.44–4.51
Log likelihood			−684.3 (df = 4)		−605.2 (df = 22)		−597.6 (df = 25)	

*N* = total number of respondents; *N* cases (%) = number and prevalence of respondents with psychological distress; df = degrees of freedom; §Fixed effects from the random effects logistic regression models.; Model 1: Adjustment for sex and age.; Model 2: + occupation and education level.; Model 3: + marital status and children under 18 living at home.

## Data Availability

Statistics Norway has an established policy for data sharing. Requests for data (i.e., The Norwegian Survey on Living conditions—working conditions) can be addressed to the Norwegian Centre for Research Data (https://nsd.no/).
